# The influence of DC voltage on the conductivity of chloroprene rubber-carbon black composites for flexible resistive heating elements†

**DOI:** 10.1039/d3ra01558k

**Published:** 2023-06-12

**Authors:** Astrida Berzina, Igors Klemenoks, Maris Knite

**Affiliations:** a Institute of Technical Physics, Faculty of Materials Science and Applied Chemistry, Riga Technical University 7 Paula Valdena Street LV-1048 Riga Latvia astrida.berzina_1@rtu.lv

## Abstract

In order to acquire a flexible resistive heating element in the temperature range for human body heating, the influence of DC voltage on chloroprene rubber (CR) and carbon black (CB) composites has been investigated. Three conduction mechanisms have been found to occur in the range from 0.5 V to 10 V – charge velocity increase due to the increase of the electric field, matrix thermal expansion that results in decreased tunnelling currents and new electroconductive channel formation at voltages above 7.5 V, where the temperature exceeds the matrix's softening point. As opposed to external heating, during resistive heating, the composite exhibits a negative temperature coefficient of resistivity up to an applied voltage of 5 V. The intrinsic electro-chemical matrix properties play an important role in the overall resistivity of the composite. The material shows cyclical stability when repeatedly applying a voltage of 5 V and can be used as a human body heating element.

## Abstract 2

Three mechanisms are responsible for the electrical conductivity in chloroprene rubber and carbon black composites for voltages up to 10 V.

## Graphical abstract

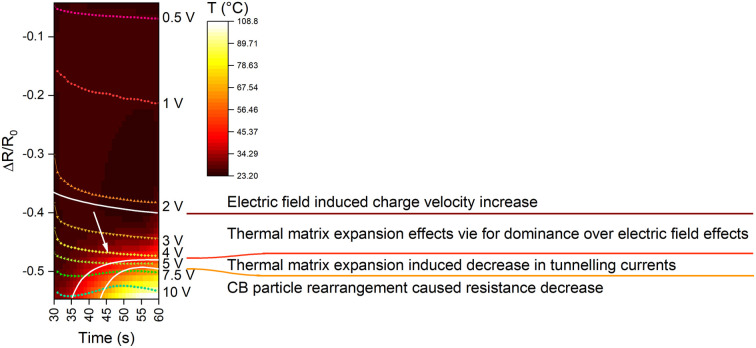

## Introduction

The global energy crisis has placed efficient use of electricity and heat back in the centre of attention. The need for protection against the elements is one of the basic human needs, so warmth is especially important in the colder months. To effectively utilise the energy for heating, one of the improvements could be heating elements that are placed close to the body and heat it directly, for example, materials that are incorporated into fabrics for clothes. The heating elements need to be elastic, preferably implemented without bulky control electronics, and safe for prolonged contact with human skin. The flexible polymer composite field is one of the most promising directions for the development of such materials.

The resistance of electroconductive polymer composites might either increase or decrease with an increase in temperature. Usually, composites with a percolative structure – composed of conductive filler particles dispersed in an insulating polymer matrix – exhibit a positive temperature coefficient of resistivity (PTC).^1–3^ It is most commonly explained by the difference in linear thermal expansion coefficients between the matrix and filler particles.^4^ The matrix expands more rapidly than the filler particles – the distance between adjacent particles increases and the tunnelling currents between those decrease.^3,5–7^ Usually, the electrical properties of the matrix are not considered for the effect, only the thermal expansion and/or crystallinity of the polymer are.

The opposite of PTC is the negative temperature coefficient of resistivity (NTC). Usually this occurs after the material reaches the melting temperature and the polymer chains and filler particles are able to rearrange themselves into new electroconductive channels.^8,9^

Chloroprene rubber (CR) or neoprene is an elastic material that is safe for prolonged contact with human skin as it has been widely used as a diving suit material.^10^

Pristine CR matrix electrical conductivity dependence on temperature for crosslinked and non-crosslinked samples^11^ proves that the electrical conductivity in CR is mainly due to an ionic conductivity mechanism. If the temperature was increased above 80 °C, then the electrical current through the sample increased with the increase in temperature (NTC). It is explained by the increase in ion mobility due to the temperature increase. The crosslinking induces additional impurities (C–Cl bond scission and ZnCl_2_ impurity formation from the released Cl atoms with the crosslinking agent ZnO), so the electrical conductivity is further increased.^11^ The authors estimate that the energy needed to create ion pairs in the sample is very small (266 cal mol^−1^; 111.9 J mol^−1^) compared to the chemical bond energy of ZnCl_2_ (76.4 kcal mol^−1^; 319.4 kJ mol^−1^) so this suggests that the conductive ions are the chlorine ions, which have not reacted with ZnO during vulcanization, and the impurity ions.^11^

Additionally, local parts of the CR chain can have conjugated electrons, which in conjunction with the impurities (ZnCl_2_, MgCl_2_) as “dopants”, can add some charge transport to pristine CR matrix conductivity.^12–14^ As the conjugated parts are localized, it will never be the dominant conduction mechanism.

Another research^15^ looks at CR–CB composite percolation properties and temperature dependence. For concentrations above the percolation threshold, the electrical conductivity firstly drops (PTC), but then, with an increase in the temperature, it increases (NTC). Current–voltage characteristic curves taken at different temperatures showed ohmic-conductivity for concentrations below the percolation threshold for all evaluated temperatures, but for strong applied fields, the current proportionality to voltage becomes quadratic, which the authors claim to be due to space charge limiting current. The findings from this research are a bit bizarre, as it does not mention the possibility of the sample heating up due to the high concentration of CB and strong applied electric field.

In the case of CR composites with carbon fibre, the composites with longer fibres show a lower percolation threshold in comparison with composites with shorter fibres. If the external temperature is increased for these composites, the resistivity also increases (PTC). It is explained with different linear thermal expansion coefficients for the filler and matrix, which means a decrease in tunnelling currents if the temperature is increased.^16^

As can be seen from previous works with CR composites, only the ambient temperature change or voltage is considered, but for an application as a resistive heating element, the influence of internally heating with an applied voltage is more important as those are the working conditions.

In this work, we investigate the possibility of developing heating elements from CR–CB composites. Special emphasis is put on the influence of the electrical properties of the matrix on the overall thermo–electric properties of this composite material. The main goal is a flexible heating element that doesn't require bulky electronics but can be easily powered from a USB port (5 V) and heats up to 40–60 °C, so it can be used for human body heating.

In this paper, we propose that the intrinsic properties of the matrix play an important role in the composite electrical conductivity *vs.* voltage and temperature. As opposed to previous studies, the temperature is changed by heating the material internally as a resistive heating element and not externally, so the influence of voltage and temperature differs.

## Experimental

### Materials

CR–CB composites were prepared by dispersing CB electroconductive nanoparticles (Printex XE-2, Degussa) in CR (Baypren 611 MV 43 ± 6 MU, Arlanxeo) *via* a solution method in a fume hood. The CB nanoparticles have a primary particle diameter of approximately 30 nm, a DBP absorption value of 380 ml g^−1^, and a specific surface area of 950 m^2^ g^−1^. The uncured CR contents are shown in Table 1. The CR is mixed with the crosslinking agents in an industrial two-roll mixer.

**Table 1:** Uncured chloroprene rubber contents

Component	Mass parts, phr
Baypren 611 chloroprene rubber (CR)	100
Zinc oxide	5
Magnesium oxide	4
Stearic acid	1

### Sample preparation

**Fig. 1:**
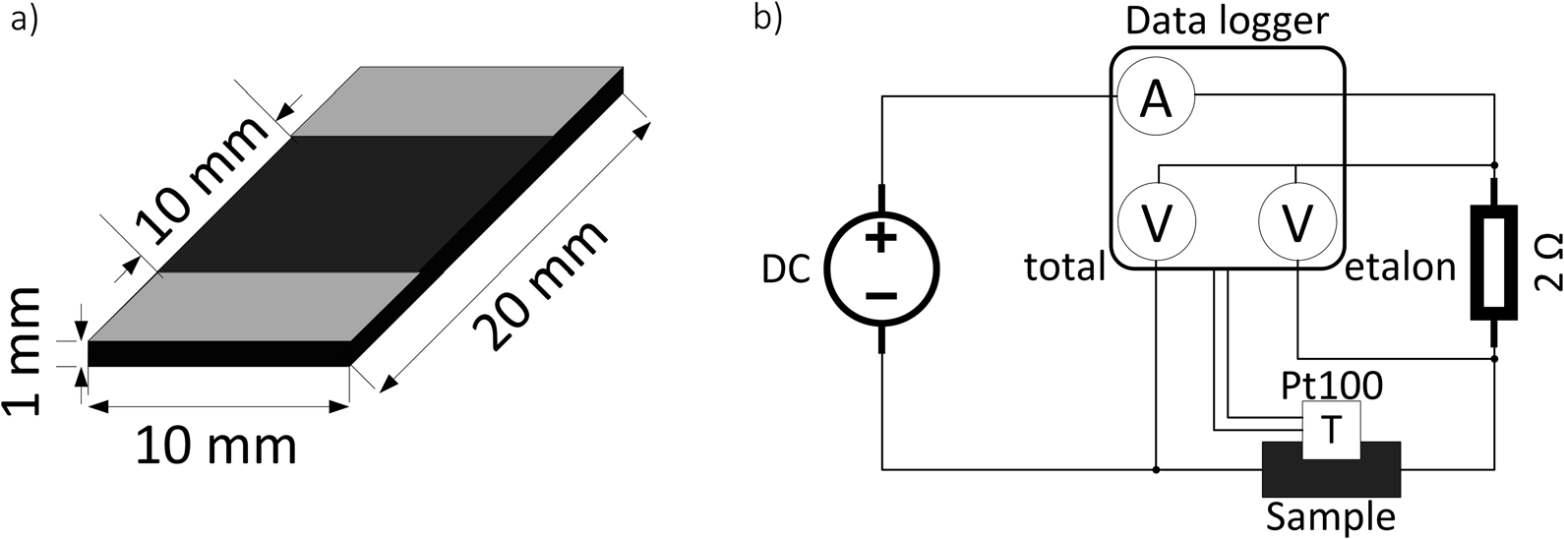
Schematic representation of sample dimensions and electrode positions (a) and electrical scheme for the resistance and temperature measurements with different applied voltages (b).

The samples are prepared by, firstly, dissolving CR into chloroform (15 ml g^−1^ CR) and, secondly, dispersing the appropriate amount of CB (10, 20 and 30 phr) in chloroform (35 ml g^−1^ CB) with an ultrasound processor (Hilscher UP200St) for 5 min with a power of 1 W ml^−1^. Afterwards, both mixtures are combined and further blended overnight on a magnetic stirrer. The mixture is then poured out in the fume hood for the solvent to evaporate. The solid compound is further homogenized on a cold two-roll mixer and cured (170 °C, 30 atm, 10 min) into 1 mm thick sheets. The samples are cut out of these sheets – 20 × 10 × 1 mm (Fig. 1a). Silver-coated copper conductive paint (MG Chemicals UK Ltd) electrodes are drawn as shown in Fig. 1a. The sample nomenclature is “CR–CB (20)” where the number in brackets denotes the degree of filling of CB in phr (parts per hundred rubber).

### Measurement setup

A typical measurement for current and temperature dependence on applied voltage consists of 30 s initial resistance measurement in constant current mode (1 mA), afterwards, a specific voltage is applied for 30 s in constant voltage mode (0.5–20 V). Then the DC power supply is switched back to constant current mode (1 mA), and the relaxation curve is measured for 9 min. The electrical scheme of the measurement setup is depicted in Fig. 1b. The resistance is calculated from the voltage drop on a high stability (0.02%) etalon resistor using the formula:1
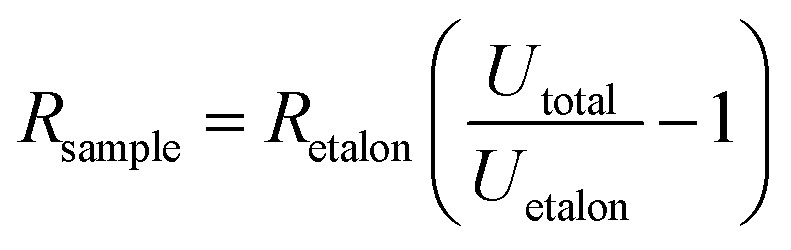


The temperature is measured with a Pt100 temperature sensor and logged with an Agilent 34972A data acquisition unit, together with current, voltage drop on the etalon resistor (*U*_etalon_), and total voltage drop on the sample and etalon resistor (*U*_total_).

Pristine matrix electrical resistance is measured with a Keysight electrometer/high resistance meter B2987A with an applied voltage of 100 V as the source. The temperature and voltage dependence measurements are done in a Faraday cage. The temperature was increased externally with a self-made electric heating element to reduce noise. First, the sample is heated from 20 to 60 °C at a rate of approximately 1.2 °C min^−1^, afterwards, the heating element is turned off and the sample is allowed to cool down naturally. The voltage dependence measurements are done with a stepwise potential increase: first, the voltage is held constant for 1 min, then the measurement point is acquired.

The linear thermal expansion coefficient (*α*) of the composite was calculated by measuring the area expansion *vs.* temperature of small composite samples while they are heated and cooled (3° min^−1^) in a precise temperature control system for microscopy (Linkam THMSE 600). The area expansion was obtained by taking a black-and-white picture of the sample every 30 s with a camera (Hikvision MV-CA020-20GC), and the sample area in pixels was calculated by summing the dark pixels (coded in MatLab). The threshold for sample/background was adjusted for each measurement series as it depends on the lighting conditions. The area of pixels (px) *vs.* temperature curve was fitted to determine the *α* of the composite material.

## Results and discussion

If a voltage is applied to a conductive polymer composite, the material starts to generate heat due to Joule heating (also called resistive heating). The overall conductivity in this case is dependent not only on the applied voltage but also on the temperature of the material, as resistance for most materials is dependent on temperature. These parameters are interrelated: the higher the applied voltage, the more heat is emitted and the higher the temperature of the material. Here we try to differentiate the influence of temperature and voltage on the material properties, therefore, the results will be divided into two sections: thermal influence (timewise long) and voltage influence (timewise short).

### Pristine CR matrix properties

**Fig. 2:**
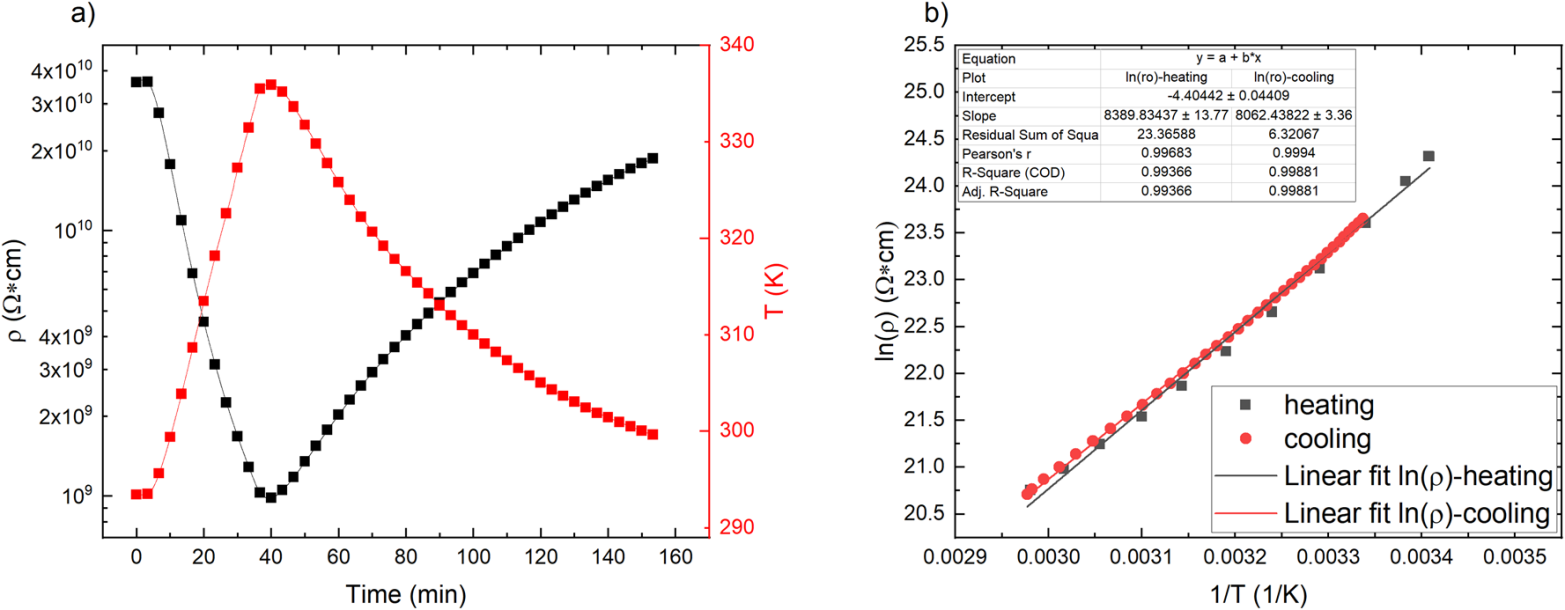
Pristine CR matrix properties: (a) resistivity and temperature change in time scale; (b) semilogarithmic plot of resistivity against reciprocal temperature for a heating/cooling cycle.

Looking at the pristine matrix electro–thermal properties (Fig. 2a), the resistance decreases with increasing temperature, which shows that there are some additional charges that contribute to the electric conductivity. The overall resistivity is rather low compared to nonpolar polymers (polyethylene > 10^15^ Ω cm^17^), which proves that polar groups, some conjugated double bonds, and impurities significantly contribute to the overall electric conductivity.^11,12,18^ The resistivity *vs.* reciprocal temperature semilogarithmic graph (Fig. 2b) shows that the thermal influence on the matrix is reversible – no significant hysteresis is observed for the cycle of heating and cooling. The exponential change follows the Arrhenius law^19^ (see Fig. 2b linear fits (S1†)): the greater the temperature, the greater the number of charge carriers that can overcome the activation energy and contribute to the conductivity.^19^

**Fig. 3:**
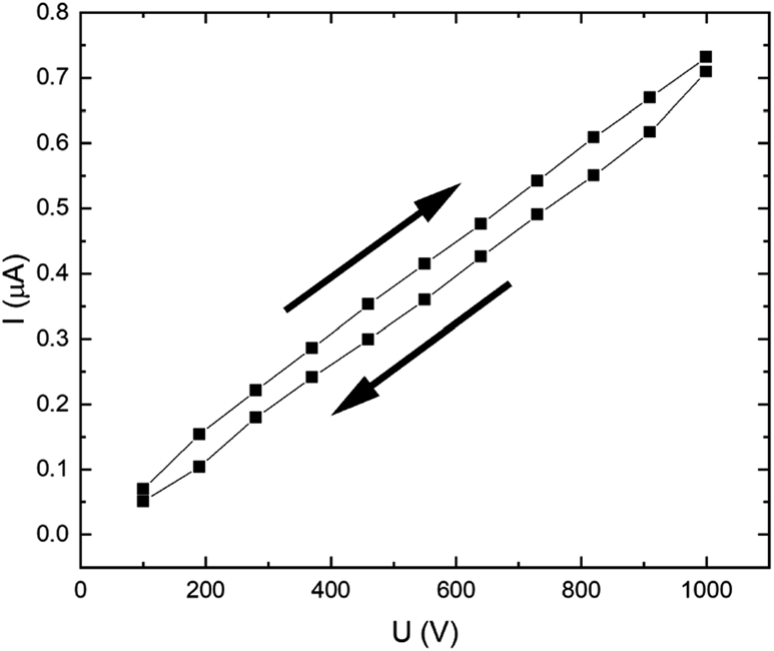
Pristine chloroprene rubber matrix current–voltage characteristic curve.

Measuring the current–voltage characteristic curve of the pristine CR matrix (Fig. 3), we can see that the character is linear throughout the applied voltage range of 100–1000 V. A small hysteresis is observable for the “to” and “from” directions, which can be attributed to some ionic impurities present in the matrix.

### Composite properties

By increasing the temperature, the polymer matrix of the sample starts to expand, which decreases the tunnelling currents between adjacent CB particles throughout the material.^3,5–7^ Also, an increase in temperature increases charge density either for the impurities in the matrix, locally conjugated electrons formed on parts of the matrix backbone, or for the charges in CB particles.^14,19^ By increasing the applied voltage, the charge velocity is increased.^20^ On the one hand, the increase of voltage and temperature increases the charge velocity and density, respectively, on the other hand, the matrix thermal expansion with increased temperature decreases the tunnelling currents – these two effects are competing for dominance on the overall electrical conductivity.

**Fig. 4:**
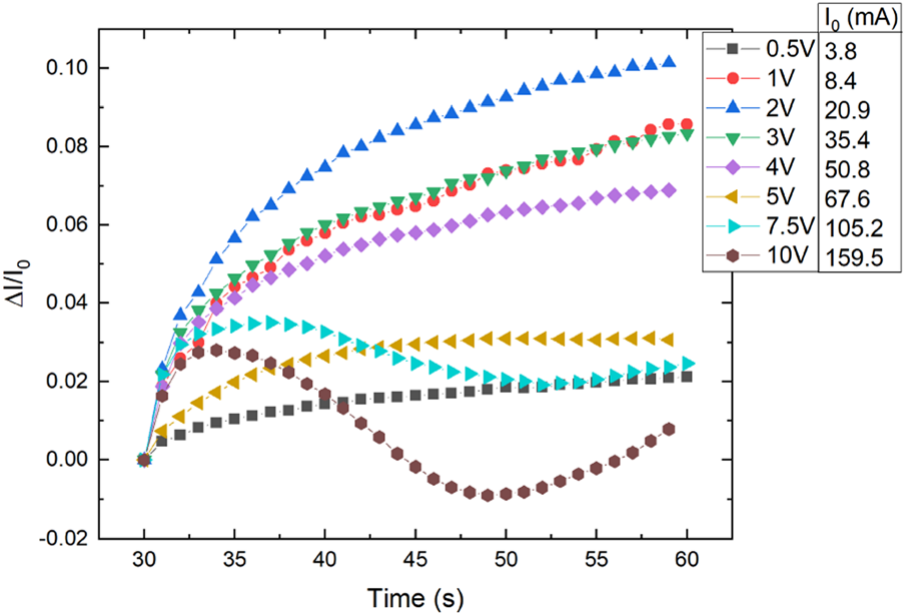
Relative current change *vs.* time for different applied voltages to CR–CB (20) samples. *I*_0_ – the initial current at the start of the applied voltage.

CR–CB composite samples with different loads of filler material were investigated at various applied voltages. A relative current *vs.* time graph for 20 phr chloroprene samples (average sample resistivity ∼130 Ω cm) with different applied voltages is shown in Fig. 4. The current increases with time for all voltages in the first couple of seconds. But a distinct difference can be seen for voltages higher than 7.5 V, where the current starts to drop after 5–7 s of an applied electrical field, with a subsequent increase at around 20–22 s after the start. The mutual influence of the two effects – voltage and temperature – can be seen here. Up to around 2 V, the dominant influence is voltage – the sample has not started to significantly heat up and expand, so it shows the highest relative current increase due only to the increase in charge velocity. Afterwards, the relative current strength decreases compared to lower voltages, but the tendency remains ascending, with further voltage increase – the material distinctly starts to heat up, and the effect of the temperature starts to dominate. At the end, the relative current strength increases again for voltages higher than 7.5 V because the temperature increases above the polymer softening point and, due to kinetic agglomeration, the electroconductive nanoparticle network is able to rearrange itself and form new electroconductive channels.^9^ According to literature, the melting temperature of uncross-linked polychloroprene varies from 45 to 75 °C.^21,22^ The softening occurs only in the parts of the uncross-linked polymer, but as the CB concentration is high and the CB particles are mobile due to their outer surface consisting of graphene sheets, only a small part needs to soften for the particles to be able to rearrange. According to our data, the temperature reached at 7.5 V is just above 72 °C, just at the melting point of uncross-linked polychloroprene.

**Fig. 5:**
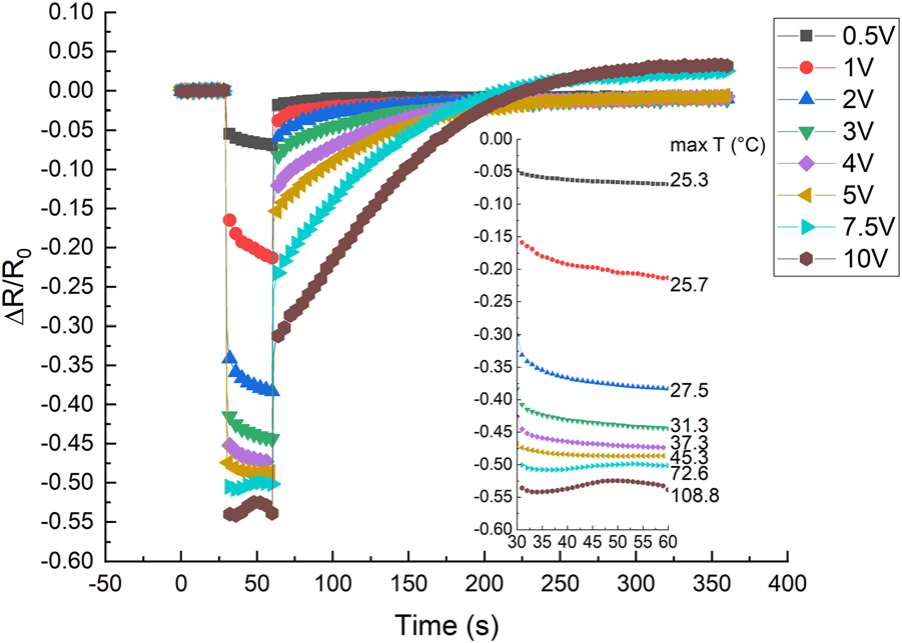
Relative electrical resistance change *vs.* time for CR–CB (20) samples. In the first 30 s, a measuring current of 1 mA is applied, afterwards, a specific voltage is applied for 30 s, and finally, a measuring current of 1 mA is applied to measure the relaxation. The inset graph shows in magnification only the 30 s part with an applied voltage, and next to it, the attained highest temperature after 30 s of an applied voltage.

In the relative resistivity *vs.* time graph (Fig. 5), we can observe the voltage influence on the electrical resistivity. For the first 30 s, a low voltage of mV was applied to measure the initial resistance, afterwards, for 30 s, the appropriate experimental voltage was applied, and subsequently, a small voltage was applied to monitor the relaxation resistance. A noticeable drop in resistance is observed after the first second when a higher voltage is applied. The initial decrease in resistance can be attributed to (i) an increase in charge velocity (*v* proportional to *E*)^20^ (ii) Maxwell–Wagner interfacial polarization effect.^23^ Due to the interfacial polarization effect, at the interface of two materials with different charge carrier relaxation times, charges accumulate if an electric field is applied.^23^ The accumulated space charge has the most influence on conductivity at the time of switching between measuring voltage (mV) and applied voltage (V) and back.^24^ This is contrary to previously determined results of nonpolar matrix composites, for example, ethylene–octene copolymer/CB composites, which show an increase in the electrical resistance with temperature, starting from the first second of an applied voltage.^25^ The insert to the plot shows an enlarged graph of only the heating period. The previously mentioned tendency is clearly visible: up to 5 V, the resistance gradually decreases, but with higher voltages (above 7.5 V), the resistance first slightly decreases but then increases with a subsequent decrease after 50 s. The later effects are due to temperature induced matrix expansion and the rearrangement of particles above the softening point, which will be discussed later.

**Fig. 6:**
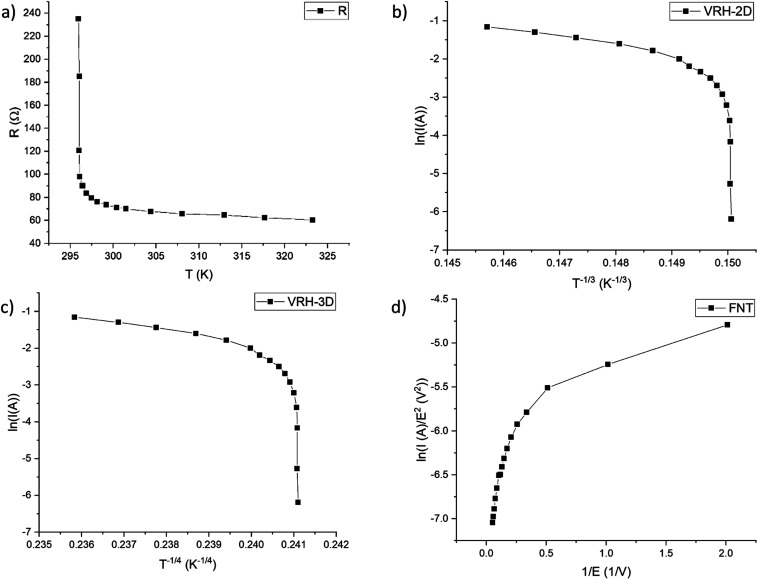
CR–CB (20) sample: (a) resistance *vs.* temperature graph; (b) semilogarithmic current *vs.* reciprocal temperature (*T*^−1/3^) and (c) *vs.* reciprocal temperature (*T*^−1/4^) for VRH 2D model and 3D model respectively; (d) FNT plot – ln(*I*/*E*^2^) *vs.* 1/*E*.

The resistance *vs.* temperature graph (Fig. 6a) shows that the material has semiconductor-like temperature dependence characteristics. The conduction mechanism is neither variable range hopping (VRH)^26–28^ as plotting the logarithm of current as a function of *T*^−1/3^ for a 2D model (Fig. 6b) or of *T*^−1/4^ for a 3D model (Fig. 6c) does not yield a linear graph nor Fowler–Nordheim tunnelling (FNT)^29,30^ for which, if plotting ln(*I*/*E*^2^) *vs.* 1/*E* (Fig. 6d), a linear graph should be observed. Our data fit very poorly to these models – all the respective specific plots show non-linear characteristics (Fig. 6). A brief model description with formulas is given in the ESI (S2).†

**Fig. 7:**
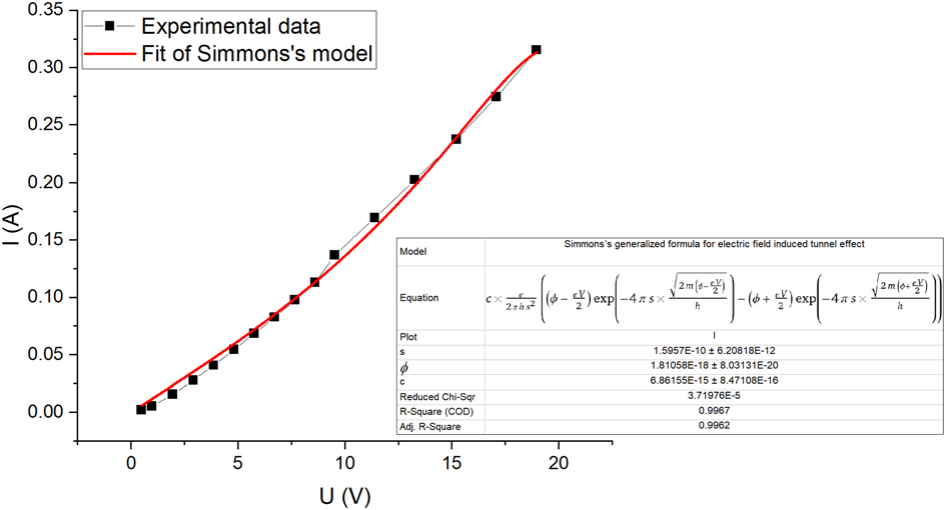
Current–voltage characteristic curve for CR–CB (20) samples. The fitted line (red) represents Simmons's generalized formula (2) for electric tunnel effect for the case of intermediate voltage (*V* < *φ*_0_/*e*).

The current–voltage characteristic curve of the composite (Fig. 7) shows a non-ohmic current dependence on voltage. This is due to electric field induced tunnelling currents, which occur between two adjacent CB particles that are separated by a thin insulating layer. The best fit to our experimental data is from Simmons's generalized formula (2) for electric field induced tunnel effect for the case of intermediate voltage (Fig. 7),^31^2
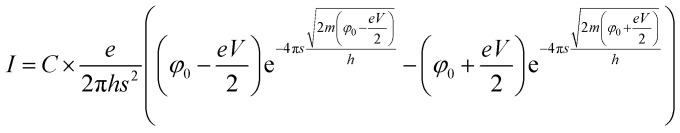
where *e* – charge of an electron, *h* – Planck's constant, *s* – thickness of the insulating film, *φ*_0_ – height of rectangular barrier, *V* – voltage across film, *m* – mass of electron and *C* – constant that is comparable to the cross-sectional area of current flow. The fitting shows that the mean cross-sectional area is about 6861.55 nm^2^ and the average layer thickness between CB particles is about 1.6 Å, which is possible with a large degree of CB filling. The potential barrier height (∼11 eV) is higher than usually considered in polymer composites (1–5 eV).^32,33^ The possible explanation could be the unusually close tunnelling distance, as these two values are considered for the tunnelling probability. The closer the distance, the higher can be the barrier for the same probability of electron tunnelling to occur.

### Thermal expansion

**Fig. 8:**
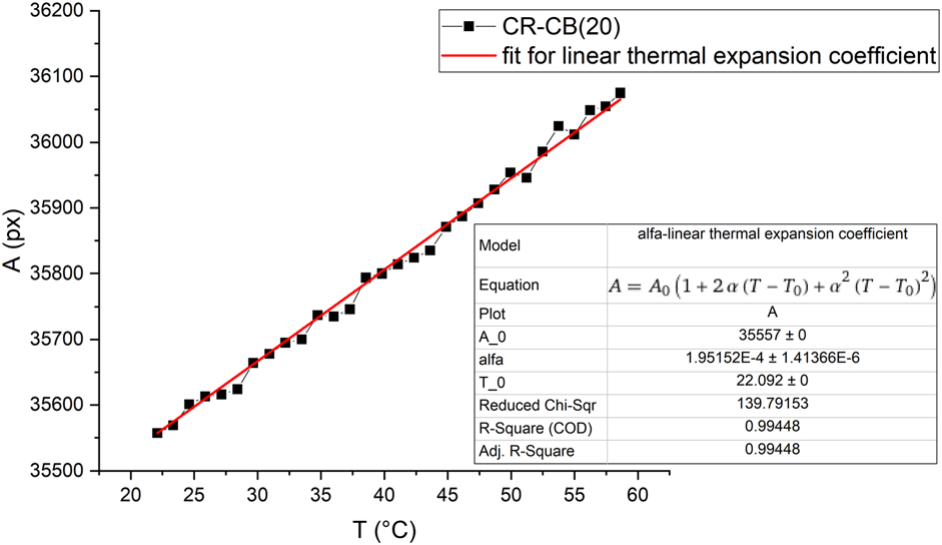
Sample pixel area *vs.* temperature graph for CR–CB (20) composites. The fit shows the linear thermal expansion coefficient *α*.

A typical area thermal expansion measurement is shown in Fig. 8. The expansion in both directions of the sample area is linear, and the calculated *α* for CR–CB (20) samples is *α* = (1.946 ± 0.104) × 10^−4^ °C^-1^. The influence of thermal expansion is seen in Fig. 5, starting already from 3 V to 5 V, where the conductivity increasing and decreasing factors are in a dynamic equilibrium. Analysing the results, the expansion with applied 5 V (at 45.3 °C) is approximately 0.39% (calculated using the measured *α*). It is already a noticeable increase in the tunnelling barrier thickness, as the effect of it can be seen in the decreased values starting from 3 V. With applied 7.5 V (at 72 °C), the expansion reaches 0.9%, which is sufficient to decrease the tunnelling currents significantly, becoming the dominant mechanism for overall conductivity, explaining the further increase in resistance in Fig. 5.

### Practical application

**Fig. 9:**
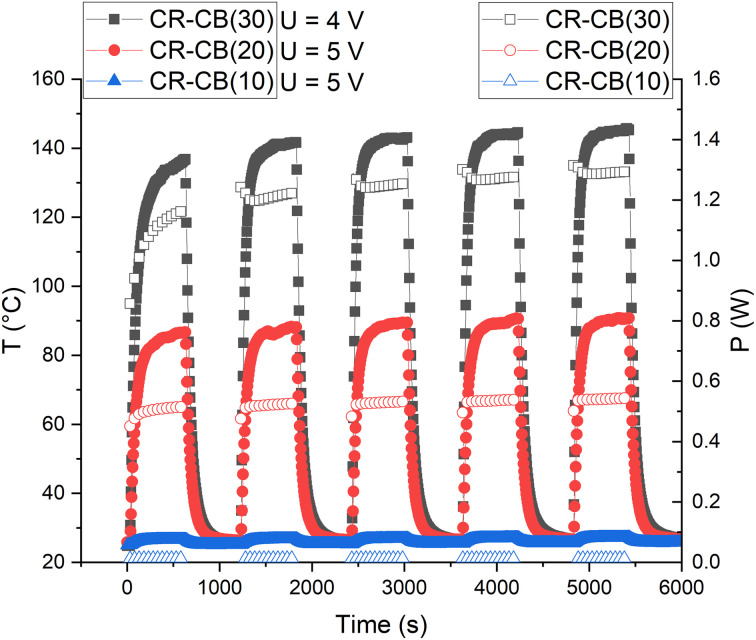
Temperature (filled markers) and output power (empty markers) of different filler load CR composites for five cycles of on/off voltage.

For practical applications as a resistive heating element, the cyclical stability of the composite is determined by applying a voltage of 5 V repeatedly (Fig. 9). After the first cycle, the CR–CB composites show remarkable cyclical stability and reversibility for temperature and output power. This only applies for voltages of up to 5 V, as higher voltages would destruct the composite. Although the generated output power increases with temperature (NTC effect), for applications where the heat is rapidly dissipated, such a powerful but elastic heating element could be useful.

## Conclusions

As opposed to nonpolar polymers, the two additional types of charge transport inherent in the CR matrix – ionic transport for charges formed from impurity ions on the matrix backbone and local regions of π-conjugated double bond charge transport on the polymer chain – play a significant role in the overall conductivity of the CR–CB composites.

Three competing conduction mechanisms have been identified in the CR–CB composites: electric field induced charge velocity increase (<2 V), thermal matrix expansion induced decrease in tunnelling currents (<7.5 V), and CB particle rearrangement because the temperature passes the softening point (>7.5 V).

Up to 2 V, only the electric field induced charge velocity increase is noticeable. At higher voltages up to 5 V, thermal matrix expansion vies for dominance with the charge velocity increase mechanism. In the region from 5 V to 7.5 V, thermal expansion is the dominant mechanism as the electrical resistance only increases. At voltages above 7.5 V, only for the very first couple of seconds does the conductivity increase, afterwards, the conductivity decreases rapidly due to the thermal expansion of the matrix and subsequent decrease in tunnelling currents. A further increase in conductivity is observed because the temperature passes the softening point and the CB particles are able to rearrange themselves, forming new electroconductive channels.

The best model for the electric field enhanced conductivity is Simmons's generalized formula for the electric tunnel effect for the case of intermediate voltage (*V* <*φ*_0_/*e*) which yielded an average barrier thickness of 1.6 Å.

The material shows cyclical stability if a voltage of 5 V is repeatedly applied and can be used as an elastic human body heating element in practical applications.

## Author contributions

A. Berzina: conceptualization, validation, formal analysis, investigation, data curation, writing—original draft preparation, visualization. I. Klemenoks: conceptualization, methodology, formal analysis, investigation, resources, writing—review and editing, supervision, project administration. M. Knite: conceptualization, methodology, formal analysis, investigation, resources, writing—review and editing, supervision, funding acquisition.

## Conflicts of interest

There are no conflicts to declare.

## Supplementary Material

RA-013-D3RA01558K-s001
