# Supplementary material for: The influence of DC voltage on the conductivity of chloroprene rubber-carbon black composites for flexible resistive heating elements
Source: RSC Adv. 2023 Jun 12;13(26):17678–84. doi: 10.1039/d3ra01558k (PMC10258886; doi:10.1039/d3ra01558k)
Supplement: RA-013-D3RA01558K-s001 [file RA-013-D3RA01558K-s001.pdf]

Electronic Supplementary Material (ESI) for RSC Advances.
This journal is © The Royal Society of Chemistry 2023

Supplementary information

S1 Arrhenius equation

Describes the conductivity for a thermally activated process[1]:

$$\sigma = \sigma_0 \, exp\left(\frac{-\Delta E_a}{k_B T}\right) \qquad \textbf{(S1)}$$

σ – conductivity, σ_0 – constant, equal to conductivity at zero Kelvin, ΔE_a – activation energy for conduction, T – absolute temperature, k_B – Boltzmann's constant.

S2 A brief conductivity model description

As NTC effect is explainable by a hopping or tunnelling mechanism of charge carriers, Mott's VRH model was considered. VHR model considers the temperature dependence on DC conductivity in amorphous and disordered materials (applicable to polymer composites). The charge carrier moves (hops) between two localized states that have similar energies, but are spatially separated. The main formula is [2]:

$$\sigma(T) = \sigma_0 exp\left[-\left(\frac{T_0}{T}\right)^{\gamma}\right]$$

where σ_0 – considered as the limiting value of conductivity at infinite temperature, T_0 – the characteristic temperature that determines the thermally activated hopping among localized states at different energies and considered as a measure of disorder, and the exponent γ is related to the dimensionality. For 2D systems $\gamma = 1/3$, but for 3D systems $\gamma = 1/4$.

FNT - Fowler-Nordheim tunnelling

This model considers that electrons are able to cross a barrier of a height due to its bending because of intense electric field. The simplified formula for the model is[3]:

$$I = CF^2 e^{-\alpha/F}$$

where I – tunnelling current strength, F – electric field, and C and α – constants.

For tunnelling in composites, the simplified formula expands [4]:

$$I \propto \sum_{1}^{n_1} v^2 exp\left(-\frac{8\pi s\sqrt{2m\phi^3}}{2.96ehv}\right)$$

where I – tunnelling current strength, v – voltage, s – thickness of insulating layer of the potential barrier, ϕ – potential barrier height, m – electron mass, h - Planck's constant.

References

1 R. Zhang, Y. Bin, R. Chen and M. Matsuo, *Polym. J. 2013 4511*, 2013, **45**, 1120–1134.
2 G. C. Psarras, *Compos. Part A Appl. Sci. Manuf.*, 2006, **37**, 1545–1553.
3 R. H. Fowler and L. Nordheim, *Proc. R. Soc. London. Ser. A, Contain. Pap. a Math. Phys. Character*, 1928, **119**, 173–181.
4 S. Pandey, C. Biswas, T. Ghosh, J. J. Bae, P. Rai, G. H. Kim, K. J. Thomas, Y. H. Lee, P. Nikolaev and S. Arepalli, *Nanoscale*, 2014, **6**, 3410–3417.
